# DEMENTIA-FOCUSED ADULT DAY CARE IN AN URBAN SETTING: PROGRAM STAKEHOLDERS’ VIEWS OF CRUCIAL COMPONENTS

**DOI:** 10.1093/geroni/igad104.2893

**Published:** 2023-12-21

**Authors:** Joy Ernst, Faith Hopp

**Affiliations:** Wayne State University, Detroit, Michigan, United States; Wayne State University, Detroit, Michigan, United States

## Abstract

Dementia focused adult day centers (ADCs) provide socialization, physical activity, and cognitive activities for participants, and respite for caregivers. This study reports findings from a process evaluation of an ADC serving a predominantly African American population in a large midwestern city. The evaluation examined whether the program was being implemented as intended, what was working/not working, and participant engagement. We conducted in-depth interviews of staff, volunteers, and student interns and surveyed caregivers (n=17). Thematic analysis of the interviews revealed themes such as “structured flexibility” in running the daily activities, an ethos of care that mixes encouragement of participation with respect for autonomy, ongoing and positive activity, and individualized attention to participants. Other findings revealed the value of consistent and experienced staff and reliance upon and benefits for students and volunteers. Quantitative and qualitative responses to caregiver surveys supported the interview themes. Caregivers valued the socialization and creative stimuli provided to participants. Guidance from program personnel included modeling communication strategies and being “in the moment” with the participants. The findings suggest that further work to inform the development of quality care and programming to improve the quality of life of older African Americans with dementia and their caregivers is needed. Specifically, there is a need to 1) identify essential components of ADCs; 2) document the ways that key ADC service philosophies are realized and communicated; and 3) explore the key role of volunteers and students in terms of their roles, activities, and motivations for working with older adults with dementia.

